# River Sediments Downstream of Villages in a Karstic Watershed Exhibited Increased Numbers and Higher Diversity of Nontuberculous Mycobacteria

**DOI:** 10.1007/s00248-023-02326-3

**Published:** 2023-12-16

**Authors:** Helena Modra, Vit Ulmann, Milan Gersl, Vladimir Babak, Ondrej Konecny, Dana Hubelova, Jan Caha, Jan Kudelka, Joseph Oliver Falkinham, Ivo Pavlik

**Affiliations:** 1 Faculty of Regional Development and International Studies, Mendel University in Brno, Zemedelska 1, 613 00 Brno, Czech Republic; 2 Public Health Institute Ostrava, Partyzanske Nam. 7, 702 00 Ostrava, Czech Republic; 3 Faculty of AgriSciences, Mendel University in Brno, Zemedelska 1, 613 00 Brno, Czech Republic; 4 Veterinary Research Institute, Hudcova 296/70, 621 00 Brno, Czech Republic; 5 Department of Biological Sciences, Virginia Tech, Blacksburg, VA 24061 USA

**Keywords:** Village impacts on water streams, *Mycobacterium avium*, *Mycobacterium fortuitum*, Wastewater treatment effluent impacts, Human activities, Mycobacterial ecology

## Abstract

The impact of residential villages on the nontuberculous mycobacteria (NTM) in streams flowing through them has not been studied in detail. Water and sediments of streams are highly susceptible to anthropogenic inputs such as surface water flows. This study investigated the impact of seven residential villages in a karst watershed on the prevalence and species spectrum of NTM in water and sediments. Higher NTM species diversity (i.e., 19 out of 28 detected) was recorded downstream of the villages and wastewater treatment plants (WWTPs) compared to sampling sites upstream (i.e., 5). Significantly, higher Zn and lower silicon concentrations were detected in sediments inside the village and downstream of the WWTP’s effluents. Higher phosphorus concentration in sediment was downstream of WWTPs compared to other sampling sites. The effluent from the WWTPs had a substantial impact on water quality parameters with significant increases in total phosphorus, anions (Cl^–^and N-NH_3_^–^), and cations (Na^+^ and K^+^). The results provide insights into NTM numbers and species diversity distribution in a karst watershed and the impact of urban areas. Although in this report the focus is on the NTM, it is likely that other water and sediment microbes will be influenced as well.

**Graphical Abstract:**

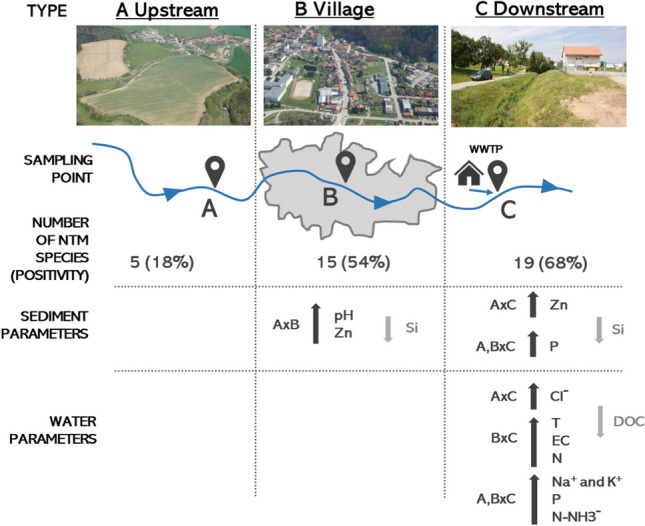

## Background

Nontuberculous mycobacteria (NTM) are widely distributed in the environment [1, 2], typically, in soil [3, 4] and dust [5] followed by surface water sediments [6], indoor water biofilms [7], surface water biofilms [8], small-scale distributed water purifiers [9], in free-living amoebae isolated from wastewater [10], and other matrices [11–14]. Due to the high surface hydrophobicity of NTM cells, NTM densities are higher in biofilms and sediments compared to water [8, 15]. NTM have an important role in degrading of organic compounds arising from anthropogenic activity [16–18] and, thus, might be found in higher numbers in villages compared to surrounding forests. Due to these characteristics, NTM can be considered “persistent bacterial indicators of environmental influences” [19, 20].

Limited information is known about the impact of small villages on NTM prevalence in surface water and water sediments in minor water courses. In this study, we focused our interest on a specific karstic watershed. Genus *Mycobacterium* was detected in karst cave environment for the first time in 1994 [21]. A karstic watershed has the following characteristics including (1) engulfed streams in karst landscape and (2) underground water channels, caves, and sinkholes due to the solubilization of limestone. These factors make karstic caves and aquifers highly vulnerable to anthropogenic contamination [22–27]. Focused on microbial contamination, special attention in karstic water has been devoted to the transport of pathogenic *Escherichia coli* and fecal indicator bacteria [28–36].

We hypothesized that water and wastewater originating from villages’ households would trigger a broader diversity of NTM spreading detected in cave sediments in one karst area (Moravian Karst, *Moravský kras*; Czech Republic). Thus, this study aimed to record the species and types NTM in karstic water and water sediments both upstream or downstream villages and treated water from wastewater treatment plants (WWTPs). In addition, we measured the physical and chemical parameters of the water and sediment samples, along with the number and species of NTM from samples collected upstream, within, and downstream of the villages and their WWTP’s outflows of seven selected villages.

## Methods

### Study Area

**Fig. 1:**
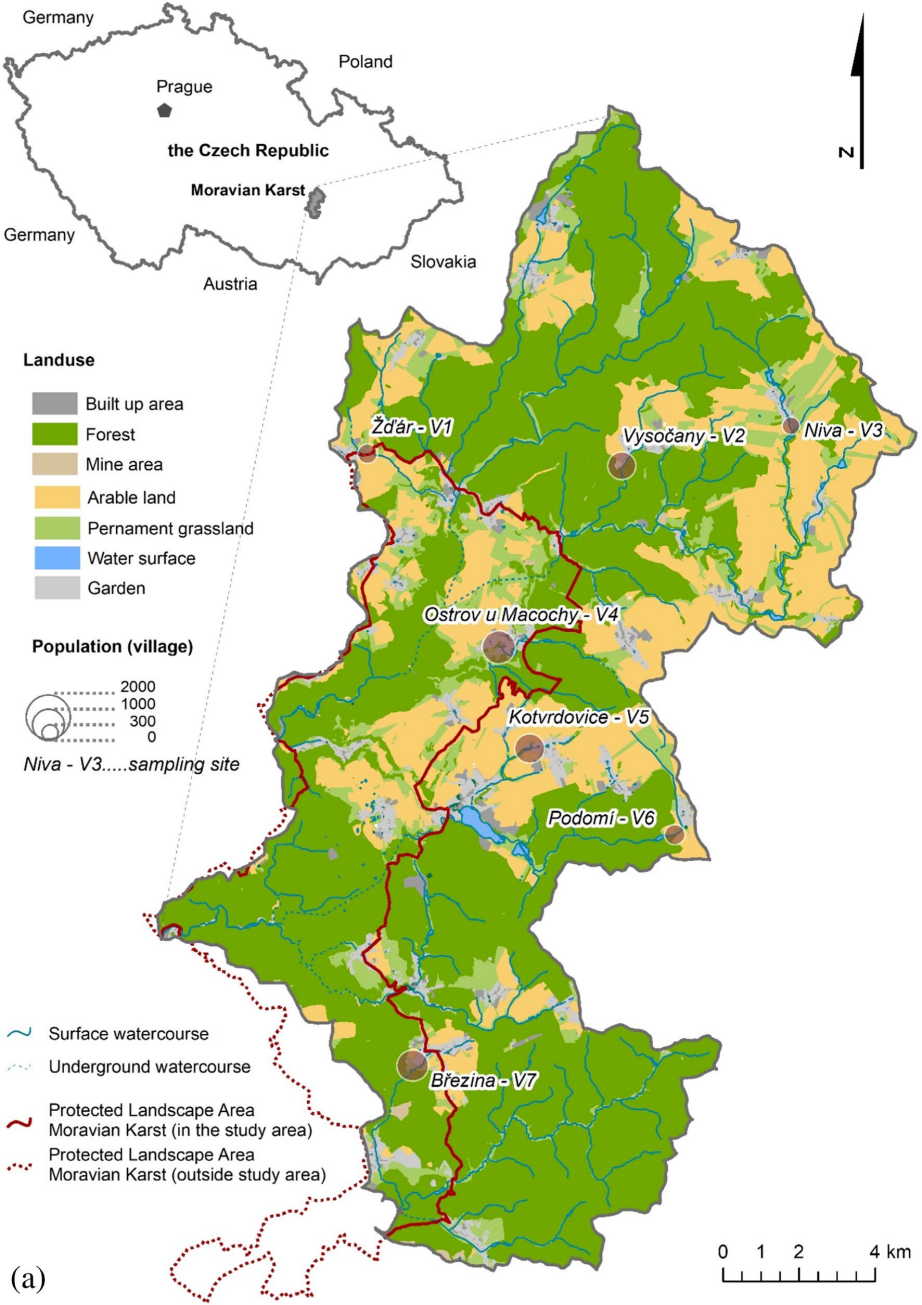
Moravian Karst (*Moravský kras*) Protected Landscape Area and its watershed with land use, sampling sites, and population sizes in seven selected villages V1–7

**Fig. 2:**
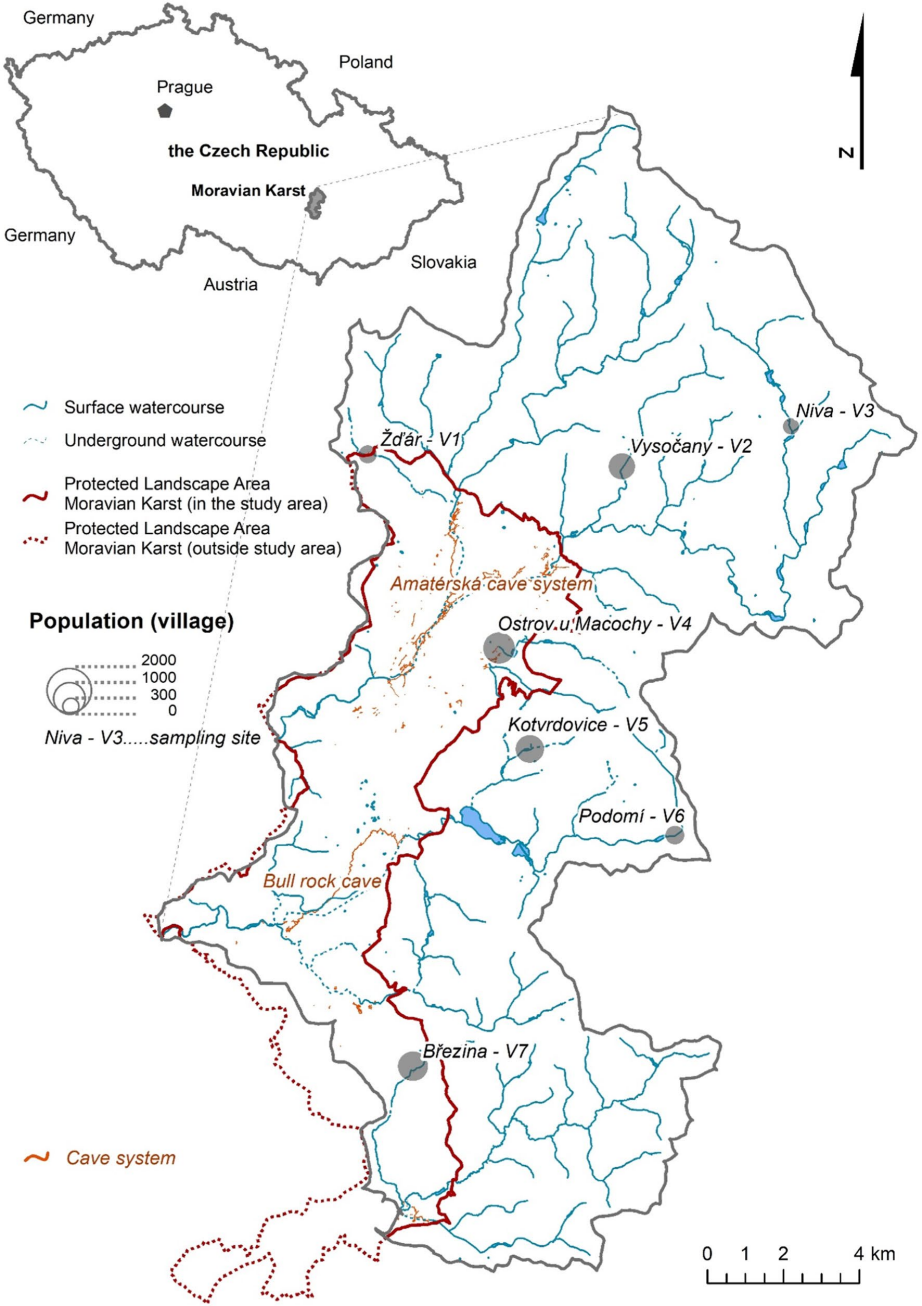
Moravian Karst (*Moravský kras*) Protected Landscape Area, its watershed with surface and underground watercourses, and with cave and cave systems, sampling sites, and population in seven selected villages V1–7

The study was carried out in the Moravian Karst (Czech Republic) with its Protected Landscape Area (PLA) of approximately 92 km^2^ established in 1956 (Fig. 1) [37] and surrounded watershed area in the southeast of the Czech Republic. That region has the two most extensive cave systems in the Czech Republic. *Amaterská jeskyně* Cave System is long; more than 34 km and cave system created by linking *Rudice* Swallow Hole (*Rudické propadání*), Bull Rock Cave (*Býčí skála*), and *Barová* Cave (*Barová jeskyně*) is long as well, than 13 km (Fig. 2) [37]. The Moravian Karst PLA is in a cold to moderately warm climatic region according to Quitt’s classification, average annual precipitation reaches 550–650 mm, and average annual air temperature ranges between 5 and 7 °C [38]. The studied area was defined by watersheds created by seven water streams: V1, *Žďárský voda* Stream; V2, *Bělička* Stream; V3, *Bílá voda* Stream; and V4, *Lopač* Stream; V5, *Kotvrdovický potok* Stream; V6, *Podoský potok* Stream; and V7, *Ochozský potok* Stream.

### Sampling Design

Sampling sites (SS) were located in seven villages (villages, V1–V7; Figs. 1 and 2) with 318–1117 population [39] based on followed criteria:

1. Samples were collected from a minor flow of seven different water streams within seven villages (average flow rate *Q* = 0.015–0.500 m^−3^/s); all water streams are connected to one river called *Svratka*, which flows outside the studied area.
2. Samples were collected in the first village on the water stream from the spring.
3. Samples were collected from parts of water streams in which chlorine compounds were used for treating drinking water used in households.
4. Samples were collected from every village treated wastewater downstream from the WWTP or other type of wastewater treatment.

Within each of the seven villages, the water and water sediments were sampled in for three different sampling sites (SS):

1. SS-A: upstream of the particular village (300–1500 m),
2. SS-B: within the confines of the village (100–300 m) up to WWTPs, and
3. SS-C: downstream of the village and the WWTP’s outflows (10–30 m downstream of the WWTP’s outflows).

Following seven villages with watercourses were sampled (Figs. 1 and 2): V1 *Žďár* (water stream: *Žďárský potok*), V2 *Vysočany* (water stream: *Bělička*), V3 *Niva* (water stream: *Bílá voda*), V4 *Ostrov u Macochy* (water stream: *Lopač*), V5 *Kotvrdovice* (water stream: *Kotvrdovický potok*), V6 *Podomí* (water stream: *Podomský potok*), and V7 *Březina* (water stream: *Ochozský potok*). The total number of sampling sites was 21.

The Czech Republic lies in the moderate climate zone with average annual spring and autumn season temperature between 1.1 and 9.7 °C depending on geographical factors. To avoid extreme temperature during the summer and winter seasons, the samples were taken during the spring (March) and the autumn (September), when the average temperature reaches 7 to 8 °C. Water from the 21 sampling sites was sampled two times per year (42 samples) during the spring and the autumn seasons (in March and September) in 2019. Water samples were collected in the water column. Submerged water sediments samples were taken four times (84 samples) at each sampling site (in March and September in the years 2018 and 2019).

Water was collected in to two 0.5-L sterile plastic disposable bottles without thiosulfate (*Radnor Township*, PA, USA). The water sediments (i.e., 30 g) were taken at depths of 0–3 cm directly into a sterile 60-mL PP sputum container (DISPOLAB s.r.o., *Troubsko*, Czech Republic) for mycobacterial investigations. The water sediments for physical and chemical analyses were taken in to the plastic storage bag in amount 200 g. Samples were transported in a cold box to laboratories and stored in a refrigerator at 6 °C in average no longer as for 24 h till the analyses. Field measurements of water parameters were not conducted upstream of the villages (SS-A) due to less reachable terrain for analytic equipment.

### Mycobacterial Culture and Isolates Identification

Water samples (1000 mL collected in to two 0.5-L sterile plastic disposable bottles) were filtered through 0.45-µm Teflon filters (Millipore, Merck, *Molsheim*, France). Each filter was transferred to a 30-mL propylene container intended for centrifugation (Medline Scientific, *Oxon*, UK) filled with 10 mL of distilled water and around of 20 pieces of glass beads (2 mm) and vortexed 30 s. The filter was discarded, and the eluate decontaminated to avoid overgrowth of the naturally slow-growing mycobacterial culture by substantially fast-growing microbial flora consisting of other common bacteria and fungi [42, 43].

Each water sediment sample (maximum of 10.0 g) was mixed with distilled water up to full volume of 30-mL plastic container (Medline Scientific, *Oxon*, UK) and vigorously shaken for 10 min to obtain maximal suspension homogeneity and to dissolve large soil clusters and release mycobacterial cells. Then, it was centrifuged for 10 min at approx. 150 × g to allow for water sedimentation of insoluble particles and most of the residual material, which could hamper the decontamination process as they interact with the chemicals and weaken their effect [44, 45]. A total of 10 mL of each turbid supernatant was transferred to a new 30-mL container and centrifuged for 20 min at 3200 × g. Pellets obtained as a final product of sediment were homogenized and decontaminated adding 10 mL of decontamination agent described below.

For water samples, a total volume of 20 mL of decontamination agent was added to 10 mL of microfilter eluate obtained previously in 30-mL polypropylene container.

Decontamination of all samples was provided by 4% NaOH (Merck, *Darmstadt*, Germany) in the 1:1 mixed solution with 1% alkyltrimethylammonium bromide (Merck, *Darmstadt*, Germany). After adding decontamination agent, mixtures were shacked using horizontal shaker (Rotamax 120, Heidolph, *Schwabach*, Germany) for 15 min. Then, the samples were centrifuged for 20 min at 3200 × g, whole supernatant was discarded, and to pellet, 15 mL of distilled water was added for neutralization. After short time (30 s) of vortexing, neutralized content was centrifuged for 20 min at 3200 × g over again. Whole supernatant was discarded, and pellet was re-suspended in 0.8 mL of physiological saline solution, as described previously [42, 43].

A total of 800 µL of suspension (re-suspended pellet) per 200 µL was inoculated in duplicate into two slants with Lowenstein-Jensen medium (in-house made) without and with sodium pyruvate. Incubation was done in parallel for 3 months at 28 °C and 37 °C. Mycobacterial growth was examined after the first week and every other week [40, 41]. Due to expected viability affecting action of decontamination agent even for mycobacteria, evaluation of loss rate/yield was provided. Using control strains of *Mycobacterium avium* DSM 44157 and *M.* *fortuitum* DMS 46621 (German Collection of Microorganisms and Cell Cultures GmbH, *Berlin*, Germany), the yield of decontamination method for water samples was estimated 78% and for solid (sediment) samples 69%.

All suspected NTM isolates were first identified by macroscopic and by microscopic (Ziehl–Neelsen staining) examinations [40, 41]. Indicative identification was done by AccuProbe Test (Hologic, Inc., San Diego, CA, USA) method, which covers only *M.* *avium* complex species, *M.* *kansasii*, and *M.* *gordonae*. Unidentified mycobacterial species by this AccuProbe Test were examined by PCR with reverse hybridization on cellulose strips GenoType *Mycobacterium* CM/AS assays (Hain Lifescience GmbH, *Nehren*, Germany), which covers additional 25 mycobacterial species. Species not identified by these hybridization methods were determined by sequencing 420 bp long region of the 16S rRNA gene (Applied Biosystems Genetic Analyzer ABI3130 series, Thermo Fisher Scientific, Inc., Waltham, MA, USA).

The DNA of mycobacterial isolates was extracted and used as template for PCR amplification of the 16srRNA and hsp65 genes using the universal bacterial primers 5′CCT ACG GGN GGC WGC AG3′ and 5′GAC TAC HVG GGT ATC TAA TCC3′ of the V3 and V4 variable regions and *Mycobacterium* hsp65 primers 5′ACC AAC GAT GGT GTG TCC AT3′ and 5′CTT GTC GAA CCG CAT ACC CT3′, respectively. Identification of mycobacterial species was performed by BLAST + (ver. 2.14.0) analysis.

### Physical and Chemical Analyses

Water parameters, temperature, pH, dissolve oxygen concentration (DOC), oxidation–reduction potential (ORP), and electrical conductivity (EC), were measured at the place and time of sampling. The temperature and pH were determined by measurement device GMH 5530 with GE 117 electrode (GHM Messtechnik GmbH, *Standorf Greisinger*, Germany). EC was measured by GMH 5450 equipment with LF 425 electrode, and DOC was determined by GMH 3630 with integrated temperature and pressure measuring. WTW inoLab Multi 720 tool with a SenTix 41 electrode (WTW Ltd, Prague, CR) was used during ORP measurements.

Chlorides and sulfates were measured by HPLC using the Dionex ICS-2000 Ion Chromatography System (ICS-2000, Thermo Fisher Scientific, Inc., Waltham, MA, USA) with IonPac® AS 18 analytical (2 × 250 mm) column. Acid neutralization capacity to pH 4.5 (ANC_4.5_) and alkalinity were determined by titration with 0.1 N hydrochloric acid. Alkalinity was performed using potentiometric titration according to the ISO 9963–1:1994 (Water quality—Determination of alkalinity—Part 1: Determination of total and composite alkalinity). Carbonate (HCO_3_^–^) concentrations were calculated based on the results of ANC_4.5_ and alkalinity. Total nitrogen was measured by chemiluminescence using Total Organic Carbon Analyzer and Total Nitrogen Measuring Unit (VCPH, Shimadzu Corporation, Japan). Individual nitrogen forms (N-NH_3_ and N-NO_3_^–^) were measured by continuous flow analysis using the Skalar San Plus (Skalar Analytical B.V., *Breda*, The Netherlands). Calcium, magnesium, sodium, potassium, iron, and phosphorus were detected by inductively coupled plasma mass spectrometry (ICP-MS) using ICP-MS Agilent Technologies 7700 (Agilent Technologies Inc., Colorado Springs, CO, USA).

ISO methods were used to measure pH, EC, total organic carbon (TOC), and total inorganic carbon (TIC) in sediments. EC and pH were measured in water extracts. Water sediment aliquots (5 g) were mixed with deionized water (1:5; AQUAL 27, AQUAL Ltd, Czech Republic), shaken for 30 min and then sedimented for 24 h. The WTW inoLab Multi 720 tool with a SenTix 41 electrode (WTW Ltd, *Prague*, Czech Republic) was used for pH measurements (ISO 10390:2005—Soil quality—Determination of pH), the multimeter WTW Multi 3320 with a TetraCon 325 electrode (ISO 11265:1994—Soil quality—Determination of the specific electrical conductivity) for EC measurements. TOC and TIC were analyzed in dry mass of sediment (drying at 60 °C to constant weight) using an Analytik Jena multi N/C 2100S meter equipped with the module HT 1300 (Analytik Jena AG, *Jena*, Germany) according to the standard method (ISO 10694:1995—Soil quality—Determination of organic and total carbon after dry combustion; elementary analysis). Calcium carbonate and *tetrasodium ethylenediaminetetraacetate tetrahydrate* (EDTA) were used for calibrations. The homogenized samples (10 g of dry sample) were weighed and incinerated at 1400 °C with the final CO_2_ concentration reported as a peak in direct proportion to total carbon (TC). Samples containing carbonates were then acidified with hydrochloric acid before being analyzed for TOC, and TIC was calculated as the difference between TC and TOC.

Total nitrogen was measured by the Dumas combustion method using a DUMATHERM Analyzer (C. Gerhardt GmbH & Co. KG, *Koenigswinter*, Germany). NO_3_ and N-NH_3_ were extracted from samples by K_2_SO_4_ and analyzed by using spectrophotometry (N-NO_3_, 324 nm; N-NH_3_, 655 nm). Microelements (aluminum, arsenic, cadmium, calcium, copper, chromium, iron, lead, manganese, nickel, phosphorus, potassium, silicon, sulfur, and zinc) were analyzed by X-ray analysis using XRF Innov-X Systems, Inc. Value 0.25 × 1*σ* was added in the case of value lower than the limit of detection (LOD).

### Statistical Analysis

Data of physicochemical parameters of water and water sediments were evaluated using one-way analysis of variance in repeated measurements design (RM ANOVA) with within-subject factor season (spring and autumn), factor sampling site (SS-A, SS-B, and SS-C), and subject sampling villages (V1–7). Measurements taken at the same place (same village V and same sampling site SS) in different seasons (spring and autumn) were considered dependent. RM ANOVA for each physicochemical parameter was performed on the raw data, then residual analysis was performed, and if the residuals significantly violated the assumptions of homogeneity and normality, the data were logarithmized (data of sulfur were transformed using Cox-Box transformation with parameter *λ* =  − 2.32); subsequently, the RM ANOVA was repeated on the transformed data. If the RM ANOVA showed a statistically significant effect of the sampling site factor, subsequent testing was performed using Tukey’s multiple comparison test. Based on results of this test, we determined between which sampling sites there is a statistically significant difference.

The alpha-diversity of NTM sp. and ssp. isolated from three sampling sites (SS-A, SS-B, and SS-C) samples were assessed using the Shannon’s diversity index H [46]. The Hutcheson test was used to compare H-indexes according to the origin of the samples. The beta-diversity of NTM sp., ssp., and complexes isolated from these three sampling site samples (SS-A, SS-B, and SS-C) was assessed by paired PERMANOVA (Bray–Curtis dissimilarity, 9999 permutations).

Fisher’s exact test was performed to determine whether there were significant differences in NTM positivity (counts of positive/negative NTM detection) between different locality sites.

Data analysis was performed using statistical software Statistica 13.2 (StatSoft Inc., Tulsa, OK, USA) and GraphPad Prism 5.04 (GraphPad Software Inc., San Diego, CA, USA) and R-project 4.1 (packages vegan and stats; https://www.r-project.org). *P*-values less than 0.05 were considered statistically significant.

## Results

### Mycobacteria in Water Samples

NTM were rarely recovered from water samples. Only 3 (7.1%) out of a total of 42 water samples yielded five NTM species (from two samples, two different NTM were isolated) belonging to three sp. and one complex: *M. terrae* (1 isolate)*, M. avium* complex (1 isolate), *M.* *fortuitum* (2 isolates), *M.* *septicum* (1 isolate), and *M. terrae* (1 isolate). NTM were cultured only from the water samples collected in three localities (V5–7) from sampling sites SS-C collected within the village and downstream of the WWTP’s outflows (Table 1).

**Table 1:**
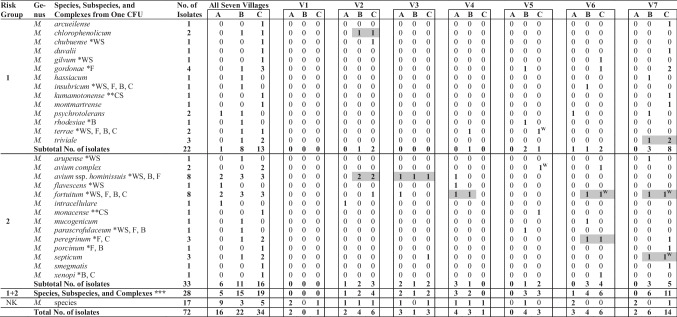
Isolated nontuberculous mycobacteria from 42 water (two repeated collections) and 84 sediment samples (four repeated collections) collected in 7 villages (V1–7) from three sampling sites A, B, and C

*M.* = *Mycobacterium*; Risk Group 1 of Agents (low individual and community risk) = includes those microorganisms, bacteria, fungi, viruses, and parasites, which are unlikely to cause disease in healthy workers or animals); Risk Group 2 of Agents (moderate individual risk, limited community risk) = includes pathogens that can cause human or animal disease but under normal circumstances, is unlikely to be a serious hazard to healthy laboratory workers, the community, livestock, or the environment according to the European Union Directive 2000/54/EC (Risk Groups are taken from LPSN https://lpsn.dsmz.de/) [68]; CFU = colony forming unit; V1–7 = villages 1–7 (V1 = *Žďár*, water stream: *Žďárský potok*; V2 = *Vysočany*, water stream: *Bělička*; V3 = *Niva*, water stream: *Bílá voda*; V4 = *Ostrov u Macochy*, water stream: *Lopač*; V5 = *Kotvrdovice*, water stream: *Kotvrdovický potok*; V6 = *Podomí*, water stream: *Podomský potok*; V7 = *Březina*, water stream: *Ochozský potok*), A, B, and C = sampling sites: A = upstream (300–1500 m) the village, B = inside the village (100–300 m) up to wastewater treatment plants (WWTPs), and C = downstream the outflow of WWTPs (10–30 m downstream of the WWTP’s outflow); ^**W**^ water samples were positive only; NK = not known; *found in Bull Rock Cave (*Býčí skála*) located in Moravian Karst (*Moravský kras*) in previous study by Modra et al. [47]: WS = water sediments and/or alluvial material, F = bats and/or earthworms in the cave, B = inside the village up to WWTPs in *Rudice* and *Jedovnice*, C = downstream the outflow of WWTPs in *Rudice* and *Jedovnice*, **found in *Amaterská* Cave System located in Moravian Karst in previous study by Modra et al. [70]: CS = cave sediment contaminated by dripping water; numbers of isolates in gray color are showing the species or subspecies isolation from different sampling sites in the same village by Pavlik et al. [48]; ***identified NTM isolates into the species, subspecies, and complexes.

### Mycobacteria in Sediment Samples

Compared to the water samples, sediment samples yielded more NTM; specifically, 48.8% of the 41 water sediments yielded NTM. There were no statistically significant differences in NTM yields between different sampling sites: 50.0% upstream (SS-A), 42.9% inside the village and upstream to the outflow of the WWTPs (SS-B), and 53.6% downstream of the village’s WWTP’s outflows (SS-C). A total of 28 NTM isolates representing different species, subspecies, or complexes were identified. *M. fortuitum* (8 isolates) and *M. avium* ssp. *hominissuis* (8 isolates) were the most frequently detected NTM found in all sampling sites. A total of 17 isolates were identified only to genus level by PCR method; further identification by sequencing due to indolent contamination by another microbiota was not possible (Table 1).

### Mycobacteria Species Diversity in Water and Sediment Samples

The NTM species diversity was significantly lower (*p* < 0.01; Hutcheson test for Shannon’s H index) in samples collected upstream of the villages (SS-A) with the detection of only five NTM species and subspecies (7 isolates): *M.* *avium* ssp. *hominissuis* (2 isolates)*, M. intracellulare* (1 isolate), *M.* *flavescens* (1 isolate)*, M. fortuitum* (2 isolates), and *M.* *psychrotolerans* (1 isolate) compared to sampling sites within the villages to the WWTPs (SS-B; 15 NTM sp. and ssp., 19 isolates) and sampling sites within the village, and downstream of the WWTP’s outflows (SS-C; 19 NTM sp. and ssp., 29 isolates). The NTM positivity was significantly higher in sampling sites within the village and downstream of the WWTP’s outflows in comparison to upstream of the villages (Fisher’s exact test, *p* < 0.001). NTM positivity did not differ with the village and downstream of the WWTP’s outflows. Six NTM species were widely distributed throughout the streams; namely, *M.* *avium* ssp. *hominissuis*, *M.* *chlorophenolicum*, *M.* *fortuitum*, *M.* *peregrinum*, *M.* *septicum*, and *M. triviale*. They were isolated from samples collected from different sites in the same village: the numbers of isolates (gray color) showing these sp. and ssp. isolates from other sampling sites in the same village. The studies reported here agree with prior studies of NTM prevalence and species diversity. Specifically, in the Moravian Karst region studied here, two previous studies reported NTM species in Bull Rock Cave (*Býčí skála*) and *Amaterská* Cave System [47, 48].

### Water Physiochemical Characteristics

Field measurements showed lower water sample oxygen concentrations (*p* < 0.01; ANOVA *F*-test) and higher conductivity (*p* < 0.05; ANOVA *F*-test) and temperature (*p* < 0.01; ANOVA *F*-test) within the village and downstream of the WWTP’s outflows (SS-C) compared to the water in the village upstream of the WWTPs in SS-B. All these parameters measured in field also statistically differed (*p* < 0.01; ANOVA *F*-test) depending on season (Table 2).

**Table 2:** Physicochemical parameters of 42 water (two repeated collections) samples

	Season							Sampling site						Season		
Parameter	log	SS	Season	x SS		Village		SS-A (*n* = 14)		SS-B (*n* = 14)		SS-C (*n* = 14)		Spring (*n* = 21)		Autumn (*n* = 21)
					Physicochemical characteristics of water from field measurement											
Temperature (°C)	No	0.0023	< 0.0001	0.0235		0.6697		n.s		6.84 ± 2.89		8.11 ± 2.13		5.24 ± 1.54		9.71 ± 0.81
DOC (mg/L)	No	0.0010	0.0062	0.2600		0.4421		n.s		9.88 ± 1.54		7.41 ± 1.93		9.56 ± 2.34		7.74 ± 1.45
ORP (mV)	No	0.9518	0.4528	0.5851		0.8073		n.s		232.43 ± 15.90		233.00 ± 36.01		237.36 ± 34.72		228.07 ± 17.24
EC (µS/cm)	Yes	0.0391	0.0075	0.9466		0.0047		n.s		361.71 ± 155.51		608.79 ± 289.90		424.86 ± 264.04		545.64 ± 252.04
					Physicochemical parameters of water											
ANC_4.5_	Yes	0.5387	0.0392	0.5152		0.6232			3.37 ± 1.40		3.42 ± 1.19		3.80 ± 1.36		3.16 ± 1.44	3.90 ± 1.07
Hydrocarbonates (mg/L)	Yes	0.4319	0.0831	0.3023		0.4564			201.29 ± 87.99		198.57 ± 68.54		231.79 ± 83.26		193.76 ± 89.14	227.33 ± 67.30
Chlorides (mg/L)	Yes	0.0041	0.6286	0.6155		0.3932			24.11 ± 12.01^a^		36.49 ± 18.84^ab^		67.07 ± 42.32^b^		42.92 ± 32.48	42.19 ± 33.44
Sulphates (mg/L)	Yes	0.6547	0.0322	0.7672		0.2994			53.57 ± 28.89		63.43 ± 58.69		64.64 ± 28.80		74.62 ± 49.55	46.48 ± 22.39
N-NH_3_ (mg/L)	Yes	0.0022	0.2813	0.3910		0.4872			0.11 ± 0.12^a^		0.20 ± 0.25^a^		2.12 ± 3.92^b^		0.27 ± 0.35	1.35 ± 3.34
N-NO_3_^–^ (mg/L)	Yes	0.2592	0.2581	0.0390		0.2502			2.90 ± 2.93		1.31 ± 1.61		3.33 ± 4.43		2.41 ± 3.59	2.61 ± 2.95
Phosphorus (mg/L)	Yes	< 0.0001	0.3274	0.8008		0.9373			0.29 ± 0.30^a^		0.77 ± 1.25^a^		2.31 ± 2.14^b^		0.81 ± 0.97	1.44 ± 2.11
Calcium (mg/L)	No	0.6830	0.4201	0.0800		0.2481			71.69 ± 33.37		70.09 ± 34.13		61.63 ± 24.64		64.30 ± 32.17	71.31 ± 29.26
Iron (mg/L)	Yes	0.0581	0.5575	0.4617		0.8386			0.42 ± 0.60		0.56 ± 0.42		1.35 ± 2.07		0.83 ± 1.22	0.72 ± 1.41
Potassium (mg/L)	Yes	< 0.0001	0.8902	0.0634		0.1793			3.78 ± 1.47^a^		6.33 ± 4.61^a^		14.93 ± 6.42^b^		8.99 ± 7.57	7.71 ± 5.62
Magnesium (mg/L)	No	0.1602	0.2386	0.6789		0.7890			9.42 ± 3.46		12.34 ± 5.56		11.72 ± 4.12		12.08 ± 5.03	10.24 ± 3.89
Sodium (mg/L)	Yes	< 0.0001	0.7019	0.5140		0.7671			18.18 ± 7.99^a^		26.58 ± 12.81^a^		59.11 ± 34.57^b^		36.30 ± 28.70	32.94 ± 27.38
Total nitrogen (mg/L)	Yes	0.0080	0.1604	0.8217		0.7429			3.71 ± 2.45^ab^		2.51 ± 1.68^a^		7.25 ± 6.21^b^		3.73 ± 4.05	5.25 ± 4.65

Physicochemical characteristics of water from field measurement in SS-B = sampling site B in the village up to wastewater treatment plants (WWTPs) and SS-C = sampling site C in the village downstream the WWTPs, (*n* = 14, mean ± SD). Significant differences by RM ANOVA, *F*-test are marked in bold. Log = a logarithmic transformation of the data was used (yes/no); SS = *p*-values of the factor sampling site (RM ANOVA, *F*-test); Season = *p*-values of the factor season (RM ANOVA, *F*-test); Season x SS = *p*-values of the interaction season x SS (RM ANOVA, *F*-test); Village = *p*-values of the factor village (RM ANOVA, *F*-test); SS-A, SS-B, SS-C = means ± SD for sampling sites; spring, autumn = means ± SD for sampling seasons; n.s. = not sampled.

*DOC* dissolved oxygen concentration, *ORP* oxidation–reduction potential, *EC* electrical conductivity.

Significantly higher (*p* < 0.05; Tukey’s test) concentrations of total nitrogen were downstream of the WWTP’s outflows (SS-C) compared to upstream (SS-B). Higher ammonia (*p* < 0.05; Tukey’s test), potassium (*p* < 0.05; Tukey’s test), phosphorus (*p* < 0.05; Tukey’s test), and sodium (*p* < 0.05; Tukey’s test) concentrations were found in water under the village, downstream of the WWTP’s outflows (SS-C) compared to the other two sampling sites upstream of the villages (SS-A) and in the village down to WWTPs (SS-B). Higher chlorides concentration (*p* < 0.05; Tukey’s test) was in SS-C compared to SS-A. No differences (*p* > 0.05; ANOVA *F*-test) were found between all three sampling sites (SS-A, SS-B, and SS-C) in the rest of physicochemical parameters of water. The results of two physicochemical parameters of water measured in laboratory (ANC4.5 and sulfates) differed depending on season (*p* < 0.05; ANOVA *F*-test) (Table 2).

### Sediment Physiochemical Characteristics

Sediments showed statistically significantly higher pH (*p* < 0.05; Tukey’s test) in the village samples up to WWTPs (SS-B) compared to samples collected within the village, downstream of the WWTP’s outflows (SS-C), and upstream (SS-A). Concentrations of calcium, copper, phosphorus, and zinc in sediments collected within the village and downstream of the WWTP’s outflows (SS-C) were significantly higher (*p* < 0.05; Tukey’s test) than other sampling sites SS-A and SS-B (Table 3).

**Table 3:** Physicochemical parameters of 84 water sediment samples (four repeated collections) in seven villages (V1–7) from three sampling sites A, B, and C

	Season					Sampling site			Season	
Parameter	log	SS	Season	x SS	Village	SS-A (*n* = 14)	SS-B (*n* = 14)	SS-C (*n* = 14)	Spring (*n* = 21)	Autumn (*n* = 21)
Ash	No	0.5746	0.6148	0.8897	0.3844	45.95 ± 15.20	54.27 ± 17.87	53.17 ± 27.56	52.78 ± 20.85	49.48 ± 20.97
Conductivity	Yes	0.4392	0.9488	0.7001	0.2916	154.53 ± 49.76	206.72 ± 159.59	357.07 ± 401.28	258.32 ± 310.81	220.56 ± 202.40
pH	No	0.0221	0.6428	0.2401	0.3539	6.44 ± 0.35^a^	6.78 ± 0.20^b^	6.69 ± 0.28^ab^	6.61 ± 0.39	6.65 ± 0.21
TC	Yes	0.2802	0.1827	0.1947	0.1449	9.61 ± 12.76	4.87 ± 2.48	5.61 ± 4.22	7.18 ± 10.98	6.22 ± 3.06
TOC	Yes	0.1841	0.0535	0.3061	0.1014	7.42 ± 7.44	3.96 ± 1.59	4.73 ± 3.55	5.19 ± 6.54	5.56 ± 2.76
Total nitrogen (%)	Yes	0.2455	0.0770	0.1176	< 0.0001	0.27 ± 0.15	0.15 ± 0.08	0.21 ± 0.14	0.20 ± 0.14	0.22 ± 0.13
N-NH_3_ (mg/kg)	Yes	0.4851	0.7473	0.2390	< 0.0001	22.82 ± 10.65	18.31 ± 13.51	18.94 ± 16.35	21.01 ± 14.37	19.03 ± 12.89
N-NO_3_^–^ (mg/kg)	No	0.6403	0.2855	0.7129	0.0020	12.67 ± 5.14	14.47 ± 7.62	15.62 ± 5.80	14.93 ± 7.28	13.57 ± 5.09
Phosphorus (µg/kg)	Yes	< 0.0001	0.5670	0.7003	0.0039	221.91 ± 73.66^a^	403.32 ± 316.11^a^	1175.7 ± 646.2^b^	556.76 ± 480.17	643.86 ± 681.57
Calcium (mg/kg)	Yes	0.1143	0.1067	0.4406	0.0032	3185.5 ± 1488.5	6911.7 ± 6289.6	7928.1 ± 5473.7	5716.7 ± 5820.0	6300.1 ± 4615.8
Iron (mg/kg)	No	0.5722	0.7820	0.6163	< 0.0001	26,484 ± 4420	24,160 ± 2203	25,371 ± 5140	25,256 ± 4177	25,421 ± 4179
Aluminum (mg/kg)	No	0.5571	0.7326	0.7282	< 0.0001	44,197 ± 4410	41,923 ± 3740	42,435 ± 3889	42,928 ± 3992	42,775 ± 4196
Silicon (mg/kg)	Yes	0.0087	0.2954	0.8100	0.0031	227,990 ± 11967^a^	219,922 ± 14664^b^	222,632 ± 25146^b^	221,576 ± 19,110	225,454 ± 17,158
Sulphur (µg/kg)	Cb	0.2116	0.2022	0.9778	0.0472	217.68 ± 299.65	202.24 ± 141.10	409.23 ± 549.24	217.03 ± 270.35	335.73 ± 453.34
Potassium (mg/kg)	No	0.2915	0.9308	0.5171	0.2069	15,859 ± 1356	15,603 ± 1066	16,511 ± 1608	16,008 ± 1386	15,974 ± 1414
Chromium (µg/kg)	Yes	0.8313	0.9013	0.7812	0.0912	32.46 ± 4.44	32.33 ± 9.04	33.11 ± 4.27	32.85 ± 7.19	32.41 ± 5.12
Manganese (mg/kg)	No	0.6751	0.3561	0.1020	< 0.0001	1206.2 ± 727.1	929.1 ± 357.3	1194.0 ± 788.2	1142.7 ± 739.8	1076.8 ± 561.5
Nickel (µg/kg)	No	0.0759	0.9672	0.2276	0.0248	19.85 ± 4.08	14.53 ± 4.79	17.32 ± 5.21	17.25 ± 5.95	17.21 ± 4.23
Copper (µg/kg)	No	0.0482	0.0233	0.6510	< 0.0001	17.12 ± 4.51	25.22 ± 9.27	26.36 ± 6.29	22.04 ± 8.62	23.75 ± 7.37
Zinc (µg/kg)	Yes	0.0062	0.5573	0.3881	0.0001	101.57 ± 19.30^a^	156.77 ± 53.97^b^	151.22 ± 27.90^b^	134.50 ± 43.60	138.54 ± 45.00
Arsenic (µg/kg)	No	0.2570	0.6384	0.3562	0.0001	15.16 ± 3.44	12.59 ± 2.58	12.31 ± 4.48	13.50 ± 4.23	13.21 ± 3.25
Cadmium (µg/kg)	Yes	0.9083	0.6935	0.7204	0.0015	13.75 ± 2.83	13.28 ± 2.27	13.70 ± 2.02	13.68 ± 2.53	13.47 ± 2.21
Lead (µg/kg)	No	0.4674	0.8978	0.1001	< 0.0001	30.73 ± 4.21	29.22 ± 5.50	26.40 ± 9.04	28.73 ± 7.59	28.83 ± 5.77

Physicochemical parameters of water sediments and results of statistical analysis. Significant differences (*p* < 0.05) between localities are marked by different alphabetic superscripts. SS-A = sampling site upstream (300–1500 m) the village, SS-B = sampling site inside the village (100–300 m) up to wastewater treatment plants (WWTPs), and SS-C = sampling site downstream the outflow of WWTPs (10–30 m downstream of the WWTPs outflow). *n* = 14; log = a logarithmic transformation of the data was used (yes/no); CB = Cox-Box transformation was used (*λ* =  − 2.32); SS = *p*-values of the factor sampling site (RM ANOVA, *F*-test); season = *p*-values of the factor Season (RM ANOVA, *F*-test); season x SS = *p*-values of the interaction season x SS (RM ANOVA, *F*-test); village = *p*-values of the factor village (RM ANOVA, *F*-test); SS-A, SS-B, SS-C = means ± SD for sampling sites; spring, autumn = means ± SD for sampling seasons.

*TC* total carbon, *TOC* total organic carbon

Lower (*p* < 0.05; Tukey’s test) silicon concentrations were found in the water sediments in SS-B and SS-C compared to sampling site upstream the villages (SS-A). Zn concentrations in SS-B and SS-C were statistically higher (*p* < 0.05; Tukey’s test) than its concentration upstream the villages (SS-A). No statistically significant differences were recorded in the rest of chemical parameters in water sediments collected in all sampling sites. The physicochemical characteristics of sediments were not dependent on season except for copper (Table 3).

## Discussion

### Water as Transport Medium of Mycobacteria

The relatively low frequency of NTM in water samples (7.1%) compared to sediments (48.8%) suggests that particulate associated NTM cells are the major sources of pathogenic NTM. These data are in accordance with previously published studies [8, 50–53]. Drinking water reservoirs with biofilms and sediments, therefore, represent a significant source of NTM [54–56]. The range of sample types did not allow us to determine the individual contributions of NTM isolates in samples collected in villages or downstream of villages and WWTPs. Both village surface water runoff and sewage plant effluents will enter the streams and contribute to both numbers and diversity of NTM.

The chlorine and disinfectant resistance of members of the genus *Mycobacterium* [57] permit survival and proliferation of NTM in drinking water distribution systems [58]. Biofilms in any water system, whether in nature or engineered systems, are the primary habitats of NTM [7, 59]. Most of the NTM detected in sediments were cultured from samples collected inside the village (*n* = 22; SS-B) or downstream of the WWTP’s outflows (*n* = 29; SS-C) sediments in the contrast to the samples collected upstream the village (*n* = 16; SS-A; Table 1). The source of these NTM could be explained by reported NTM abundance in drinking water and household plumbing systems [7, 11, 59, 60].

E.g., in the Hawaiian Islands, NTM detection from home plumbing systems was significantly higher than NTM detection from outdoor environmental water biofilms [7]. In other studies, they also found that biofilms in drinking water pipes are also richly colonized by various NTM species [59, 60].

### Villages Affected NTM Species Diversity

Both surface runoff and sewage plant outflows associated with villages had a positive effect on NTM species diversity. Possibly, the introduction of water from those sources plays an important role in NTM spreading in surface water environment, as it was published previously [63]. This was found in our study also. Significantly higher (*p* < 0.05 at least PERMANOVA) numbers of NTM species and subspecies in water sediments in the villages up to WWTPs (SS-B) and under the village, downstream of the WWTP’s outflows (SS-C) in comparison with sampling sites above village, were detected (SS-A; Table 1). Higher NTM sp. and ssp. diversity in these ecological niches (SS-B and SS-C) could also be related with higher temperature caused by wastewater from households (mentioned above) and WWTPs [64–67].

Although we did not focus our study on WWTP’s technologies, we have found that wastewater (sampling sites SS-B and SS-C) had affected NTM diversity, which was the same in these both polluted sampling sites but was statistically significantly lower in non-polluted water sediments upstream of the villages (SS-A; Table 1). Considering the proposed use of treated wastewater (i.e., reuse water), the findings here of NTM in WWTP’s effluents need to be taken into consideration. Recycling wastewater for human, animal, or agricultural production might relate to the increase of NTM due to their ability to survive disinfection [57].

### Prevalence of Mycobacteria and Human Health Risk: Risk Group 1 of Biological Agents

The largest number of 99 (51.0%) sp. and ssp. is present in the Risk Group 1 of biological agents (that are unlikely to cause human disease); they are rarely associated with disease. In clinical laboratories, these mycobacterial species are isolated from clinical samples (sputum, tissue, urine, etc.) without clinical relevance [69]. A similar situation was documented in the Czech Republic [41]. In our study, we isolated 14 sp. and ssp. from this Risk Group 1 (Table 1).

The spectrum of NTM spp. varies widely depending upon the source of the environmental samples in different locations in the Czech Republic and the material sampled: bat guano, earthworm feces, woody material, soil, etc. Of the 14 NTM sp. mentioned above in this study, only 6 sp. were demonstrated in other localities: *M.* *duvalii*, *M.* *gordonae*, *M.* *hassiacum*, *M.* *kumamotonense*, *M.* *terrae*, and *M.* *triviale* [41]. The remaining 8 sp. (*M.* *arcueilense*, *M.* *chlorophenolicum*, *M.* *chubuense*, *M.* *gilvum*, *M.* *insubricum*, *M.* *montmartrense*, *M.* *psychrotolerans*, and *M.* *rhodesiae*) were found only in this karstic watershed (Table 1). In the previous study in the Moravian Karst, four of these NTM were already proven; in the Bull Rock Cave, *M.* *chubuense*, *M.* *gilvum*, *M.* *insubricum*, and *M.* *rhodesiae* were detected [47]. The last four NTM (*M.* *arcueilense*, *M.* *chlorophenolicum*, *M.* *montmartrense*, and *M.* *psychrotolerans*) were isolated in this watershed for the first time (Table 1) [47, 70].

In *Hranice* Karst (Czech Republic; CR), 80 km from the Moravian Karst, eight NTM sp. and one complex were cultured. *M.* *arupense*, *M.* *avium*, *M.* *florentinum*, *M.* *gordonae*, *M.* *intracellulare*, *M.* *mucogenicum*, *M.* *sediminis*, and *M.* *avium* complexes were isolated from sediments in *Hranice* Abyss and *Zbrašov* Aragonite Caves [68]. Except of *M.* *florentinum* and *M.* *sediminis*, all other six NTM sp. were detected in the current study in Moravian Karst (Table 1); these two species remain unique in Hranice Karst [48].

The exact geochemical parameters and conditions for colonizing of these substrates by environmental NTM are not yet known and explained in the published literature. Therefore, it is necessary to consider that people who live in this environment are exposed also to NTM species and subspecies which are clinically irrelevant (esp. NTM included in Risk Group 2) [4, 71].

### Risk Group 2 of Biological Agents

We detected 14 sp., ssp., and complexes included in the Risk Group 2 of biological agents (Table 1), which can cause human disease (it is unlikely to spread to the community, and there is usually effective prophylaxis or treatment available) [71]. In this Risk Group 2, there are 87 (44.9%) out of 195 validated sp. and ssp.

In the Czech Republic, between the years 2003 and 2018, a total of 79% mycobacterioses were caused in children by *M.* *avium* (included in these statistics were *M.* *avium* and *M.* *intracellulare*) [72]. In adult patients with mycobacteriosis, members of the *M.* *avium–intracellulare* complex were among the most common causative agents of infection [73]. Our preliminary data shows these infections were caused especially by *M.* *avium* ssp. *hominissuis* (unpublished data). Not surprisingly, in this study, *M.* *avium* ssp. *hominissuis* was cultured from water sediments from all three different types of samples collected in our study (Table 1). Due to this fact, water sediments could represent an infection risk for susceptible children and adults.

### Risk Group 3 of Biological Agents

We did not detect any of the members of Risk Group 3 mycobacteria they are obligate pathogens and only transiently isolated from the environment (Table 1).

### Impact of Villages on Water Pollution in Area of Our Interest (Moravian Karst)

In a just published study about Moravian Karst, our area of interest, the impact of villages on pollution by allogeneic was analyzed and confirmed [27]. We have found a similar effect of settlements in the same villages and watersheds of streams (Tables 2 and 3; Figs. 1 and 2).

Higher phosphorus concentration in wastewater connected with villages could be beneficial for NTM growth as is in accordance with the results of the study published previously [74] where *Mycobacterium* spp. genes in reclaimed systems positively correlated with phosphorus. These findings suggest that phosphorous could be a growth- or survival-limiting nutrient for NTM. The phosphorus concentration was only one parameter statistically significantly increased in samples originating from sampling sites collected within the villages, downstream of the WWTPs outflows (SS-C). It is important to note that in our study, the spectrum of elements analyzed in water was lower than the spectrum of elements in sediments (Tables 2 and 3). However, nitrogen concentrations and its forms (ammonia and nitrates) did not correlate in water and water sediments (Table 3).

The predominance of NTM in drinking water distribution systems [75] also depends on “water age” (esp. long-time standing water in pipes or water reservoirs) and sufficient residual monochloramine in various sections of the potable water systems [59].

Only 6 (21.4%) of 28 detected NTM complex and sp. in sediments in our study (MAC, *M.* *gordonae*, *M.* *arupense*, *M.* *fortuitum*, *M.* *peregrinum*, and *M.* *septicum*) matched the 12 NTM sp. detected in ponds and water reservoirs sediments in the Czech Republic [51]. The correlations between the occurrence of NTM and environmental, climatic, water, and water sediment characteristics have been described [76, 77]. The critical factor increasing the occurrence of NTM in water and aerobic water sediments was acidification. This parameter did not affect NTM positivity in water sediments in our study because of a very narrow range of all pH values (5.83–6.96) among the studied types of sampling locations. However, pH values of sediments in both sites in investigated areas (SS-B and SS-C) were statistically significantly higher compared to upstream of the sampling sites, where there were no differences in NTM positivity. While nutrients and organic carbon concentrations have been frequently reported to influence microbial communities, we did not confirm higher nitrogen or carbon concentration in villages’ area sediments (SS-B and SS-C) as NTM nutrient factors.

Tourists often consider brooks, rivers, and adjacent areas as attractive recreational places. According to our findings, these water streams could represent a similar risk to urban recreational water [78]. The risk of infection is also posed by situations after extreme events (e.g., Hurricanes Harvey and Irma in 2017 in the USA), during which local flooding occurs. During them, various pathogenic bacteria, including representatives of the *Mycobacterium* genus belonging to the Risk Groups 1 and 2, are washed away and spread in the environment [79].

A higher population per square mile, proportion of area as surface water, evapotranspiration, and copper and sodium soil levels were described that significantly increase the risk for pulmonary disease caused by NTM in the USA [80]. Our study showed the presence of PPM (*M.* *avium* spp. *hominissuis*, MAC, *M.* *chelonae*, *M.* *fortuitum*, *M.* *intracellulare*, and *M.* *monacense*) in all types studied sampling sites, although their diversity was higher in sediments near villages (SS-B and SS-C). *M.* *fortuitum* and other NTM often be detected in wastewater and surface water in urbanized and suburbanized environments [56, 79, 91–84, 92–85].

Humane doctors are often asked by sick patients and parents of child patients with mycobacteriosis, what are the risks of their possible reinfection. Very often, various components of the environment are cited as sources of clinically relevant NTM [2, 11], including drinking water [9], water used for personal hygiene in heavy industry and collieries [40], water used for recreation [8, 13, 14], soil [3], indoor environment [7], and other environmental components. The results of this study point to the fact that even the immediate surroundings of the water stream in villages and below the villages can also be risky for these adult and child patients, including some other predisposed persons. From an ecological point of view, it can be considered a significant fact that the water environment in and below municipalities can be a source of many types of NMT and water is their vector.

## Data Availability

The datasets generated and/or analyzed during the current study are available in the Mendel University (FRDIS) repository and are attached in PDF file.

## Abbreviations

ANOVA: Analysis of variance
ANC4.5: Acid neutralization capacity to pH 4.5
CR: Czech Republic
DOC: Dissolve oxygen concentration
EC: Electrical conductivity
ESM: Environmentally saprophytic mycobacteria
EU: European Union
ICP-MS: Inductively coupled plasma mass spectrometry
LOD: Limit of detection
LPSN: List of prokaryotic names with standing in nomenclature
V: Village
M: Mycobacterium
MAC: *M. avium* Complex
MDR: Multi-drug resistance
NTM: Nontuberculous mycobacteria
OPM: Obligatory pathogenic mycobacteria
ORP: Oxidation-reduction potential
PERMANOVA: Permutational multivariate analysis of variance
PLA: Protected Landscape Area
PPM: Potentially pathogenic mycobacteria
sp.: Species
ssp.: Subspecies
SS-A: Sampling site A
TC: Total carbon
TIC: Total inorganic carbon
TOC: Total organic carbon
TRBA: Technical Rules for Biological Agents
WGS: Whole genome sequencing
WWTPs: Wastewater treatment plants
